# Machine learning identifies straightforward early warning rules for human Puumala hantavirus outbreaks

**DOI:** 10.1038/s41598-023-30596-x

**Published:** 2023-03-03

**Authors:** Orestis Kazasidis, Jens Jacob

**Affiliations:** Julius Kühn Institute (JKI) – Federal Research Centre for Cultivated Plants, Institute for Plant Protection in Horticulture and Forests / Institute for Epidemiology and Pathogen Diagnostics, Rodent Research, Toppheideweg 88, 48161 Münster, Germany

**Keywords:** Ecological modelling, Ecological epidemiology

## Abstract

Human Puumala virus (PUUV) infections in Germany fluctuate multi-annually, following fluctuations of the bank vole population size. We applied a transformation to the annual incidence values and established a heuristic method to develop a straightforward robust model for the binary human infection risk at the district level. The classification model was powered by a machine-learning algorithm and achieved 85% sensitivity and 71% precision, despite using only three weather parameters from the previous years as inputs, namely the soil temperature in April of two years before and in September of the previous year, and the sunshine duration in September of two years before. Moreover, we introduced the PUUV Outbreak Index that quantifies the spatial synchrony of local PUUV-outbreaks, and applied it to the seven reported outbreaks in the period 2006–2021. Finally, we used the classification model to estimate the PUUV Outbreak Index, achieving 20% maximum uncertainty.

## Introduction

Environmental conditions triggered by climate change play an ever-increasing role in the spread of zoonotic infectious diseases, by altering the animals’ natural habitats, influencing food availability and even driving changes in species distribution. Within this framework, we have developed a simple weather-based model for the human Puumala Orthohantavirus (PUUV) infection risk in Germany.

The PUUV is the most common hantavirus in Europe, transmitted by bank voles (*Clethrionomys glareolus*, syn. *Myodes glareolus*). The PUUV can cause mild-to-moderate hemorrhagic fever with renal syndrome (nephropathia epidemica) with 0.1–0.4% fatality rate^1^. The human PUUV-infections fluctuate multi-annually. Recently, there have been several years with $>{1000}$ annual reported cases in Germany^2^, generally and large-scale driven by beech (*Fagus* spec.) mast intensity, as shown in the past for Belgium^3,4^ and for Germany^5,6^.

The underlying mechanisms for the transmission rate of PUUV to humans seem too complex to model directly, as they depend on the abundance of the bank vole populations, their PUUV-prevalence, and the human-bank vole interaction; all of which fluctuate temporally and vary locally. Nevertheless, weather conditions can be used as predictors for the human PUUV-infection risk, because the fluctuation in bank vole populations strongly correlates with weather parameters from the two previous years^7^, whereas the PUUV-prevalence mainly depends on the bank vole abundance^3,8,9^.

We selected German districts with significant numbers of human infections and incidence in 2006–2021, and inspected the correlations of the annual PUUV-incidence with monthly weather parameters at the district level. We performed a data transformation that highlights the spatial synchrony of the temporal fluctuation of PUUV-incidence. This transformation allowed developing a binary classification model based on support vector machines (SVM) for the district-related outbreaks, applicable countrywide and based solely on easily-accessible weather parameters. Furthermore, we introduced the PUUV outbreak index (POI) as an indicator for the annual human PUUV-infection risk. The POI allows an unambiguous definition of a PUUV-outbreak for the first time, considering the local magnitude of the PUUV-incidence. The definition of POI is independent of the total annual infections, but their values are highly correlated. Finally, we applied the binary classification model to the POI and showed that just three values of weather parameters suffice to give a good estimate. The combined POI-model may be applied as a straightforward rule-of-thumb for the detection of high-risk years. Such a model can be used to interpret the outbreaks of PUUV, but also to get approximations about rodent dynamics. This is highly important to support strategies and decisions for the protection of human health and plants alike.

## Results

### Local outbreaks and the PUUV outbreak index

**Figure 1:**
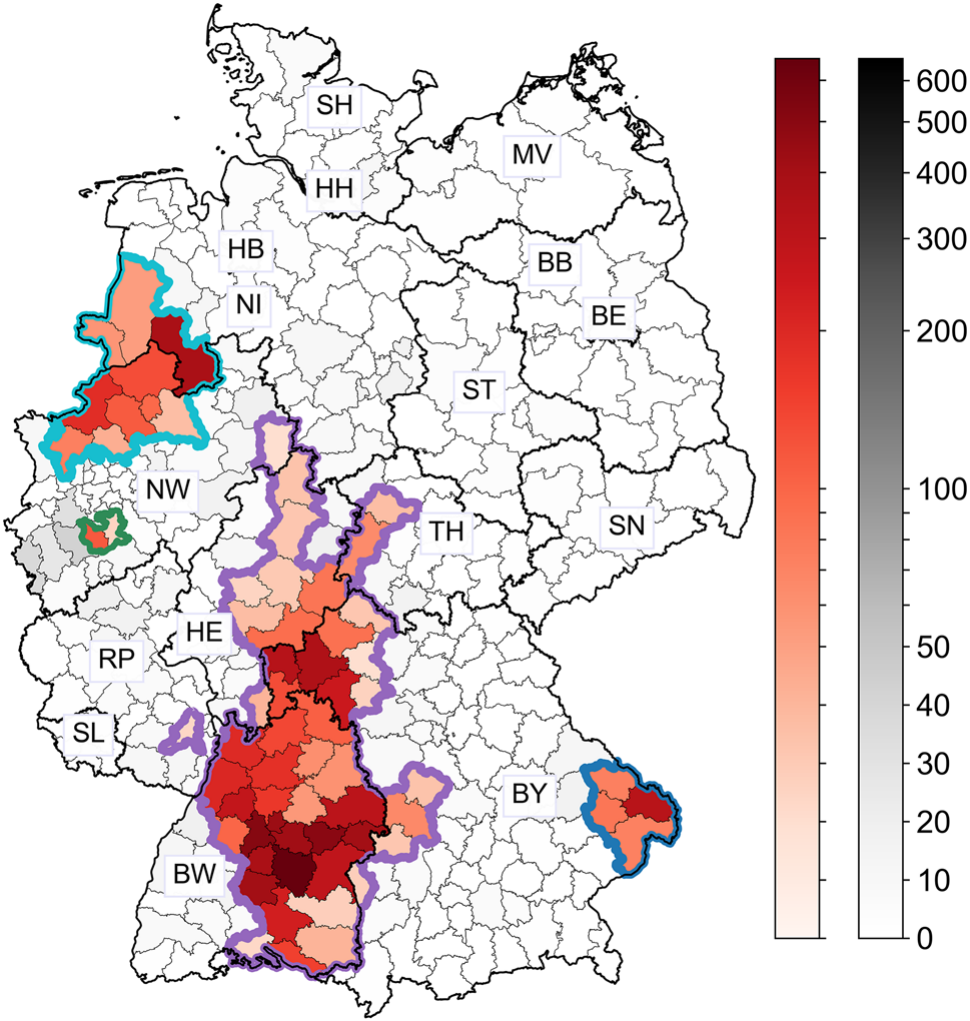
Selection of the districts for the analysis. The 66 selected districts across Germany are shown in red gradient depending on their total PUUV-infections in 2006–2021. The colorbar is linear in the range [0, 50] and log-scaled in [50, 650] for increased visibility. There were 26 districts from Baden-Württemberg (BW), 16 from Bavaria (BY), 8 from Hesse (HE), 3 from Lower Saxony (NI), 10 from North Rhine-Westphalia (NW), 1 from Rhineland-Palatinate (RP), and 2 from Thuringia (TH). Thick black lines separate the federal states; thick colored lines separate four clusters of the detected PUUV-molecular clades, as described in the text. Further districts are shown in gray gradient with the same colorbar scaling. The map was generated using the geopandas package v0.9.0 (https://geopandas.org) in Python v3.8.5. Further information about the raw data, the processing, and the visualization is provided in the Methods section.

Based on the local infection and incidence values, we selected 66 districts in Germany, where PUUV was constantly present in 2006–2021 (Fig. 1). The selected districts account for 10,090 human PUUV-infections, 89.9% of the total infections reported countrywide in this period (Fig. 1, red gradient). The districts are grouped into four clusters, agreeing with the hypothetical edge of the range of the Western bank vole evolutionary lineage^10^ and comprising all PUUV-molecular clades detected so far^11–13^. The first cluster (Fig. 1, cyan outline) contains 10 districts between Lower Saxony and North Rhine-Westphalia, at the border to the Netherlands, and corresponds to the clades of Münsterland and of the Teutoburg Forest. The second cluster (Fig. 1, green outline) comprises 2 districts in the southwest North Rhine-Westphalia, both from the clade of Rhineland. The largest cluster (Fig. 1, purple outline) contains 50 districts in a central vertical corridor with a length of about 450 km and a maximum width of about 200 km, expanding from Hesse and Thuringia, through Bavaria, until the south of Baden-Württemberg at the border to Switzerland. This cluster includes the PUUV-molecular clades of North East Essen, Spessart Forest, Swabian Jura, and Thuringian Forest. Finally, the last cluster (Fig. 1, blue outline) contains 4 districts in eastern Bavaria at the border to the Czech Republic, with the PUUV-molecular clade of the Bavarian Forest.

There were 12 districts that are combinations of an urban district with its neighboring or surrounding rural district, shown in Supplementary Table 1. The only urban districts that remained separate were Cologne (Köln), Münster, and Stuttgart, whose areas are distinctly large.

We applied a log-transformation to the incidence values, followed by an individual binary classification for each district. The resulting two classes were labelled “low-risk” and “high-risk”. A local “outbreak” occurred in a year when the incidence in a district was classified in the high-incidence bin of the recorded values. The incidence in districts for non-outbreak years was zero or considered low relative to the recorded values in this specific district. From the total 1056 observations (16 years $\times$ 66 districts), 682 were assigned low-risk (65%) and 374 were assigned high-risk (35%). A total of 8779 infections were registered in observations assigned to the high-risk class, which was 87% of the total infections included in this analysis (or 78% of the total infections in Germany in 2006–2021).

**Figure 2:**
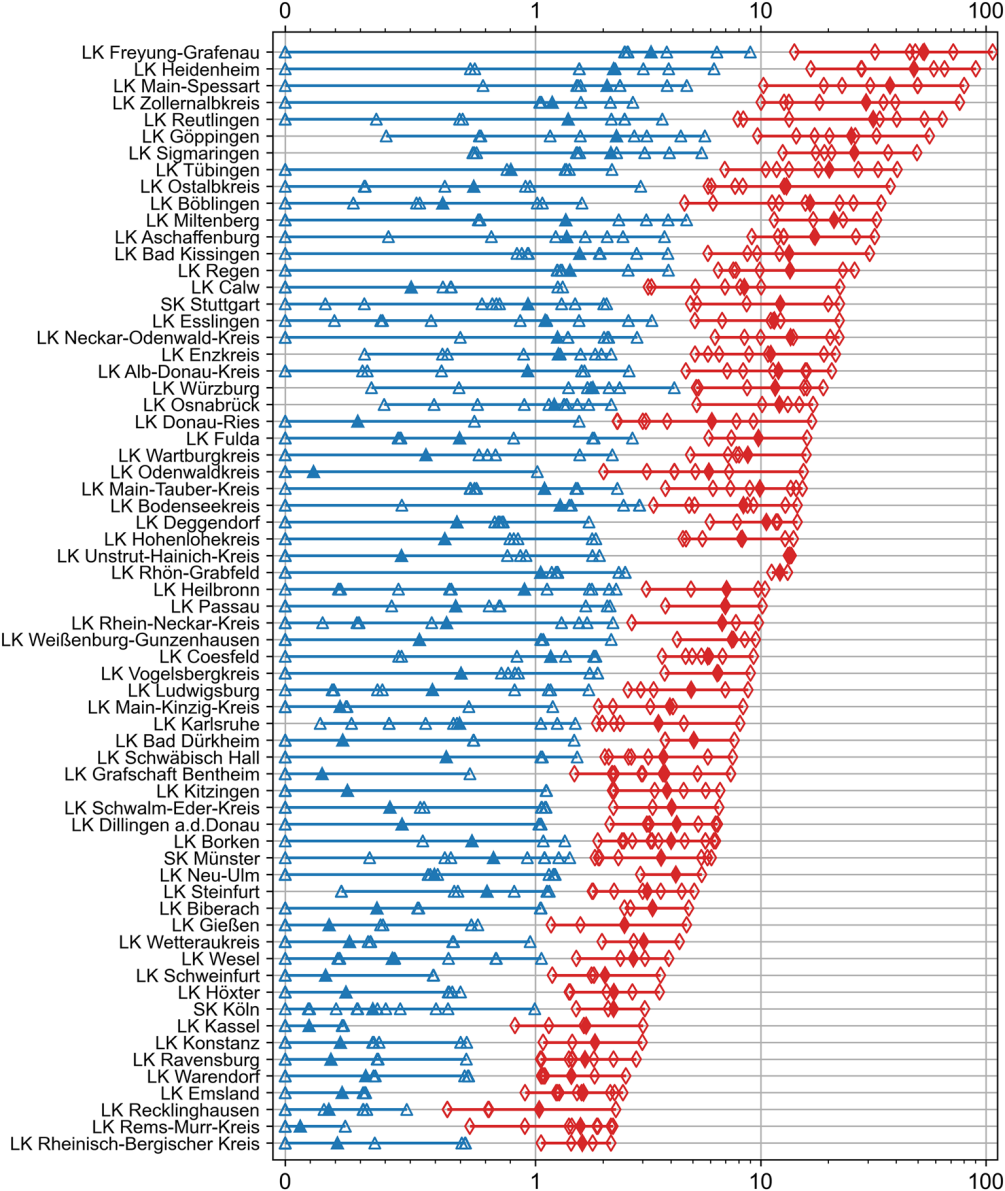
The annual incidence values in the selected districts from 2006 to 2021. The 66 districts are ordered by the maximum annual incidence. The low-risk bin is indicated by blue triangles (on the left side of the plot). The high-risk bin is indicated by red diamonds (on the right side of the plot). The filled triangles and diamonds indicate the average value for each bin. The solid lines highlight the incidence range for each bin. The white gaps between the blue and the red lines indicate the separation between the two bins for each district. The x-axis is linear in the range [0, 1] and log-scaled in [1, 110] for increased visibility. The naming convention matches that of the German version of SurvStat@RKI 2.0. LK: rural district (from the German *Landkreis*) and SK: urban district (from the German *Stadtkreis*).

As the binary classification was district-based, the same incidence value may be assigned to low risk or high risk, depending on the district (Fig. 2).

Based on the local outbreaks, we developed the PUUV outbreak index (POI) as an indicator for the annual human PUUV-infection risk in Germany, i.e., for the global PUUV outbreak. Each year’s value in the POI was defined as the proportion of districts assigned high infection risk for that year (Table 1):

1 $${\text{PUUV Outbreak Index}}\left( t \right) = \frac{{{\text{Number of districts with high PUUV}} - {\text{infection risk in year}}\;t}}{{{\text{Number of districts where the PUUV is present}}}}$$

**Table 1:** The number of districts assigned high risk for each year from 2006 to 2021.

Year	Number of districts with high risk	PUUV outbreak index—POI (%)	Total infections
2006	0	0.0	48
2007	46	69.7	1503
2008	5	7.6	157
2009	1	1.5	123
2010	59	89.4	1686
2011	6	9.1	198
2012	61	92.4	2121
2013	2	3.0	88
2014	15	22.7	285
2015	27	40.9	476
2016	6	9.1	111
2017	52	78.8	1013
2018	6	9.1	110
2019	44	66.7	918
2020	5	7.6	124
2021	39	59.1	1129

The PUUV outbreak index (POI) was calculated by dividing by the total number of districts, i.e., 66. The POI values highly correlate with the total annual infections in the selected districts given in the last column, with 0.95 Pearson correlation coefficient and a *p* value of 1.15 × 10^−8^.

Years with $>{900}$ total reported infections in the selected districts have a POI value of $>\text{50\%}$.

### Classification model

Our initial predictors’ pool comprised monthly weather parameters from the two previous years. We selected the triple of variables that led to the optimal classification model for the binarized log-transformed incidence: the soil temperature in April of two years before (V2_ST_4), the total sunshine duration in September of two years before (V2_SD_9), and the soil temperature in September of the previous year (V1_ST_9). The resulting model had 82.6% accuracy, 84.8% sensitivity, 71.4% precision, 81.4% specificity, and 0.775 F_1_-score. The elements of the confusion matrix were: true negatives ${\text{TN}}={555}$ (53% of the total 1056 observations), false negatives ${\text{FN}}={57}$ (5%), false positives ${\text{FP}}={127}$ (12%), and true positives ${\text{TP}}={317}$ (30%). 5/7 classifications for high risk were correct (precision), and almost 6/7 real high-risk observations were correctly classified (sensitivity). The observations in false negatives summed up to 301 infections, which was 3.5% of the infections in real high-risk observations (or 3.0% of the total infections).

**Figure 3:**
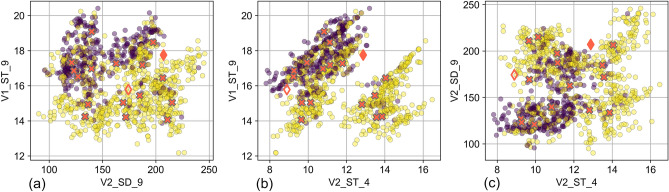
Views of the model. 2D scatter plots with all 1056 observations from 2006 to 2021 for the three pairs of variables in the selected 3D model. V1_ST_9 in (**a**) and (**b**): the mean soil temperature in September of the previous year, V2_SD_9 in (**a**) and (**c**): the total sunshine duration in September of two years before, and V2_ST_4 in (**b**) and (**c**): the mean soil temperature in April of two years before. Yellow (hex color code #FDE725FF) corresponds to observations with low risk, whereas indigo (hex color code #440154FF) corresponds to observations with high risk. The overlaying red x-markers indicate the values of the variables from each year averaged over whole Germany, called cluster centers. The red diamond markers indicate the average values over Germany for 2022 (filled markers) and for 2023 (unfilled markers).

For the pairs (V2_SD_9, V1_ST_9) and (V2_ST_4, V1_ST_9) the two risk classes were well linearly separable, with sensitivity $>\text{77\%}$ and precision $>\text{67\%}$ (Fig. 3a,b). Weather parameters are for the most part spatially uniform. Thus, the observations from each year formed clusters in the 3D input space of our model. The values of the weather variables for these “cluster centers” were the annual average values over whole Germany.

Because of the clustering of the weather variables and the separation of the data from each year, the model classified all districts from each year in the same risk class. The only exceptions were 2006 and 2021, the years whose clusters were closest to the planar class boundary. For 2006, 58 districts were classified in the low-risk class and 8 in the high-risk class. For 2021, 40 districts were classified in the high-risk class and 26 in the low-risk class. All observations from 2007, 2010, 2012, 2015, 2017 and 2019 were classified in the high-risk class, which designated them as PUUV outbreak years; whereas all observations from 2008, 2009, 2011, 2013, 2014, 2016, 2018 and 2020 were classified in the low-risk class.

The highest annual accuracy was 98% for 2009 (1 FN). For seven additional years, an annual accuracy $>\text{90\%}$ was achieved, i.e., 2012 from the outbreak years, and 2008, 2011, 2013, 2016, 2018 and 2020 from the non-outbreak years. The lowest annual accuracy was 41% for 2015 (39 FP), followed by 65% for 2021 (12 FP and 11 FN). For 2014, the only wrong classifications were false negatives (15 FN, 77% accuracy). The highest accuracy was achieved in Baden-Württemberg (90%), and the lowest in North Rhine-Westphalia (66%) and Lower Saxony (69%). There were 6 districts from Baden-Württemberg, 2 from Bavaria, and 1 from Hesse with 100% accuracy. Another 15 districts had only one false classification (9 FP and 6 FN). The maximum numbers of false negatives came from the districts of Borken and Bentheim, with 7 FN and 6 FN, respectively. Borken also had the lowest accuracy among the districts with 44% (7 FN and 2 FP).

According to our classification model, a hyperplane separated the two risk classes. This hyperplane was a planar boundary in the 3D space:

2 $${0}{\text{.270}} \cdot {\text{V2\_ST\_4}} + {0}{\text{.0139}} \cdot {\text{V2\_SD\_9}} - {0}{\text{.549}} \cdot {\text{V1\_ST\_9}} + {4}{\text{.054}} = {0}$$

Based on this plane, we could define the binary infection risk with respect to V1_ST_9, the last weather variable that becomes available prior to prediction:

3 $$\begin{aligned} & {\text{When}}\;{\text{V}}1\_{\text{ST}}\_9 > 0.492 \cdot {\text{V}}2\_{\text{ST}}\_4 + 0.0253 \cdot {\text{V}}2\_{\text{SD}}\_9 + 7.38, \\ & \quad {\text{there}}\;{\text{is}}\;{\text{high}}\;{\text{infection}}\;{\text{risk}}\;{\text{in}}\;{\text{the}}\;{\text{district}}\;{\text{for}}\;{\text{that}}\;{\text{year}}. \\ \end{aligned}$$

4 $$\begin{aligned} & {\text{When}}\;{\text{V}}1\_{\text{ST}}\_9 < 0.492 \cdot {\text{V}}2\_{\text{ST}}\_4 + 0.0253 \cdot {\text{V}}2\_{\text{SD}}\_9 + 7.38, \\ & \quad {\text{there}}\;{\text{is}}\;{\text{low}}\;{\text{infection}}\;{\text{risk}}\;{\text{in}}\;{\text{the}}\;{\text{district}}\;{\text{for}}\;{\text{that}}\;{\text{year}}. \\ \end{aligned}$$

where we have rounded the coefficients to three significant figures. In Eqs. (2)–(4), the units for the temperatures ST are °C, and for the sunshine duration SD are hours. To minimize rounding errors, ST should have a precision of at least two decimal places and SD of at least one decimal place.

### Prediction of a PUUV outbreak year

**Figure 4:**
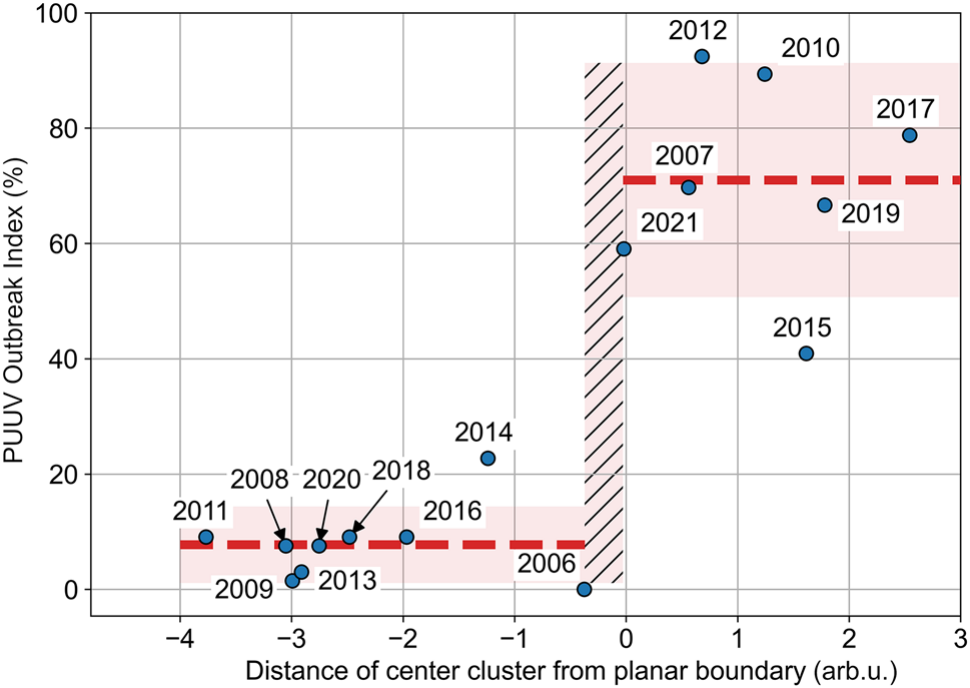
Estimating the PUUV Outbreak Index from the classification model. The proportion of districts with high risk for each year, which was defined as the PUUV Outbreak Index, is plotted with respect to the distance from the planar boundary of the cluster centers, i.e., of the points defined by the average values of the weather parameters over Germany for that year. The red dashed lines show a piecewise constant fit to the data (pseudo-R^2^ = 0.87, calculated according to^15^). The red-shaded area indicates the uncertainty. The hashed area for distances in the interval $\text{[}-\text{0.37,}-\text{0.03]}$ represents the increased uncertainty about the position of the discontinuity.

The distance of the cluster centers from the planar boundary of the classification model (Eq. 2) can serve as a qualitative measure for the global PUUV-infection risk. Figure 4 shows the POI for 2006–2021 with respect to the distance of the corresponding cluster center from the planar boundary. The observations form two groups: for ${\text{distance}}_{\text{t}}>-\text{0.03}$ (a positive distance means that the observation is above the boundary with respect to V1_ST_9; thus, there is high infection risk for that year) and for ${\text{distance}}_{\text{t}}<-\text{0.37}$ (a negative distance means that the observation is below the boundary with respect to V1_ST_9; thus, there is low infection risk for that year). We compared the groups’ means by a one-way ANOVA with the function f_oneway from the SciPy library^14^. Based on the F-value of 97 and *p* value of 1.1 × 10^−7^, we concluded that the means of the two groups were statistically significantly different.

We applied a piecewise constant function as fit; for each group we assumed a constant value for the POI, defined by the average of the recorded values of that group. The standard error of the mean was 2.21% for the low-risk group (${\text{distance}}_{\text{t}}<-\text{0.37}$), and 6.77% for the high-risk group (${\text{distance}}_{\text{t}}>-\text{0.03}$). For distances in the interval $\text{[}-\text{0.37,}-\text{0.03]}$, where the step occurred and no observation was available, no estimate could be generated. With an uncertainty equal to three times the standard error of the mean, we could estimate the POI for the year ${\text{t}}$ as:

5 $${\text{PUUV}}\;{\text{Outbreak}}\;{\text{Index}}_{t} \left( \% \right) = \left\{ {\begin{array}{*{20}l} {7.7\% \pm 6.6\% ,} & {{\text{if}}\;{\text{distance}}_{t} < - 0.37} \\ {\left( {1.1\% , 91\% } \right),} & {{\text{if}}\; - 0.37 \le {\text{distance}}_{t} \le - 0.03} \\ {71\% \pm 20\% \Rightarrow {\text{Outbreak}},} & {{\text{if}}\;{\text{distance}}_{t} > - 0.03} \\ \end{array} } \right.$$

## Discussion

We applied a rigorous and exhaustive method to select the optimal triple of weather variables for a model that predicts human PUUV infection risk. The resulting classification model had high explanatory power with almost 85% sensitivity and more than 70% precision. Although our method may not give the global maximum with respect to a specific performance criterion, it avoids including highly correlated variables and it is bound to have high sensitivity and precision. A classifier with just two variables would be more straightforward and easy to grasp. Although the addition of a third variable increases the performance only marginally, it renders the model more robust and less prone to hidden variables. By further increasing the dimensions, the separation of the two risk classes is expected to be easier, even though there is no indication that the classes are indeed perfectly linearly separable. The weather parameters from the actual year influence both the bank vole populations^16,17^ and the human activities^18^, as well as their interaction, and thus are expected to drive the reported infections in a way that cannot be encapsulated by a prediction model with variables from the previous years. Our classifier is in essence a prediction model for the beech seed production and the bank vole abundance. Therefore, it can also be applied for rodent management and plant protection strategies.

Our analysis assumes that the correlations between the weather parameters and the human PUUV-infections are the same for all districts and are time-invariant, i.e., they remain constant with time, which allows considering each observation as independent. An additional underlying assumption was that the monitoring of the hantavirus diseases and the impact of any countermeasures remain constant in each district, though they may differ among districts.

Our method reveals a strong influence of the infection risk from the weather parameters in April and September of two years before, and from the previous September. Furthermore, a weather variable from the previous September was contained in all variable pairs with the optimal performance, which places the earliest possible prediction in early October of the previous year. This should provide enough time to prepare countermeasures and to raise awareness in health authorities, risk groups and medical practitioners about the risk of the virus.

Weather variables from two years before were most likely linked to the beech seed production of the previous year, which in turn determines the food availability and governs the growth of the bank vole populations^4,6^. An increased soil temperature in autumn of the previous year could lead to a larger initial population for the next year, by facilitating the last weeks of the breeding season and increasing the rodent survival rate.

This model estimates the PUUV-infection risk, and thus it is likely to be positively biased compared to the reported infections or incidence. Therefore, we may have to accept overestimations (false positives). A close inspection of several underestimations (false negatives) is provided in Supplementary Note 1. We hypothesize that many underestimations from Lower Saxony and North Rhine-Westphalia were not due to a local outbreak, but rather were caused by an increase in the PUUV-baseline in specific districts, due to changes in the reporting system and to increased awareness in the local health departments. Another possibility is that the PUUV-season may start earlier in Northern Germany in comparison to the other PUUV-clusters. Finally, these infections may be connected with a PUUV-spread from the neighboring Netherlands.

The binarization of the incidence suggests spatial synchrony of the PUUV outbreaks in Germany. This opposes a recent report about lack of synchrony in 2019^19^, but is in good agreement with earlier studies^11,20^. The introduction of the POI allows the unambiguous definition of an outbreak year, which in turn can facilitate the transfer of prediction results through media and other public communication. A high value of the POI indicates increased risk for a large proportion of districts. This method can be easily extended to describe outbreaks of any zoonotic infectious disease with temporal fluctuation and spatial inhomogeneity.

Our classification model can be applied as a straightforward rule-of-thumb for the detection of high-risk years, although it is not strictly developed as a prediction model and is not yet validated as such. Combining it with the POI, we can predict outbreak years. This method does not offer detailed spatial information, because it uses weather parameters that form constellations, and thus can be regarded as spatially uniform across large areas. Nonetheless, such a prediction can increase the state of preparedness and raise awareness about virus detection and human infection risk. We applied this concept to estimate the PUUV-infection risk in Germany in 2022. The values for the 2022 center clusters (Fig. 3) have not been observed during 2006–2021, nor during the preceding years 2002–2005. Therefore, this year’s incidence values will allow a refinement of the model’s coefficients and decrease its uncertainty. The distance of the 2022 center cluster from the linear boundary of the model is $-\text{1.08}$, thus a low global PUUV-infection risk is expected. By applying Eq. (5), the predicted value in the POI is $\text{7.7\%}\pm \text{6.6\%}$, i.e., only about 1–10 districts are likely to report a relatively high number of infections in 2022. The distance of the 2023 center cluster from the linear boundary of the model is $-\text{0.35}$; this value falls inside the interval of increased uncertainty of Eq. (5) and does not allow a definitive estimation of the global PUUV-infection risk. Applying Eqs. (3) and (4) at the district level, 11 districts from Lower Saxony and North Rhine-Westphalia are in the high-risk class and thus are likely to report a relatively high number of infections in 2023. This leads to an expected POI of 16.7%.

Land cover and land use data have not been included in this model but have been previously reported as possible general predictors of the bank vole PUUV-prevalence^21^ and the human PUUV-incidence^17,22^. We consider that those effects are incorporated into the district-based incidence transformation, i.e., the land cover or land use may indeed determine the local magnitude of the PUUV-incidence, but they do not influence the probability of an outbreak.

In the future, this approach can be supplemented with spatial information, by including a time-variant and spatially non-uniform variable, e.g., the beech mast intensity or the beech flowering intensity as proxies for the beech seed production. Such a variable may increase the separation between the observations from 2006 and 2021, on opposite sides of the decision boundary; two years with relatively similar weather constellations but distinctly different incidence values. The years 2014 and 2015 are the outliers that do not seem to fit reasonably in the low-risk and high-risk classes, suggesting the existence of a third class with medium risk. However, the currently available observations do not suffice for distinguishing such a class.

## Methods

We performed data acquisition, processing, analysis and visualization using Python^23^ version 3.8 with the packages Numpy^24^, Pandas^25^, Geopandas^26^, Matplotlib^27^, Selenium, Beautiful Soup^28^, SciPy^14^ and scikit-learn^29^. The functions used for specific tasks are explicitly mentioned to allow validation and replication studies.

### Data acquisition and processing

#### Human PUUV-incidence

Hantavirus disease has been notifiable in Germany since 2001. The Robert Koch Institute collects anonymized data from the local and state public health departments and offers via the SurvStat application^2^ a freely available, limited version of its database for research and informative purposes. We retrieved the reported laboratory-confirmed human PUUV-infections (${\text{n}}=\text{11,228}$ from 2006 to 2021, status: 2022-02-07). From the attributes available for each case, we retrieved the finest temporal and spatial resolution, i.e., the week and the year of notification, together with the district (named “County” in the English version of the SurvStat interface).

To avoid bias through underreporting, our dataset was limited to PUUV-infections since 2006. The years 2006–2021 contain 91.9% of the total cases from 2001 to 2021. Human PUUV-incidence was calculated as the number of infections per 100,000 people, by using population data from Eurostat^30^. For each year, we used the population reported for the January 1 of that year. The population for 2020 was also used for 2021.

In the analysis, we only included districts where the total infections were $\ge {20}$ and the maximum annual incidence was $\ge {2}$ in the period 2006–2021. The spatial information about the infections provided by the SurvStat application refers to the district where the infection was reported. Therefore, in most of the cases, the reported district corresponds to the residence of the infected person, which may differ from the district of infection. To compensate partially for differences between the reported place of residence and the place of infection, we combined most of the urban districts with their surrounding rural district. The underlying assumption was that most infections reported in urban districts occurred in the neighboring or surrounding rural district. In addition, some urban and rural districts have the same health department. Supplementary Table 1 lists the combined districts.

#### Weather data

From the German Meteorological Service^31^ we retrieved grids of the following monthly weather parameters over Germany from 2004 to 2021: mean daily air temperature—Tmean, minimum daily air temperature—Tmin, and maximum daily air temperature—Tmax (all temperatures are the monthly averages of the corresponding daily values, in 2 m height above ground, in °C); total precipitation in mm—Pr, total sunshine duration in hours—SD, mean monthly soil temperature in 5 cm depth under uncovered typical soil of location in °C—ST, and soil moisture under grass and sandy loam in percent plant useable water—SM. The dataset version for Tmean, Tmin, Tmax, Pr, and SD was v1.0; for ST and SM the dataset version was 0. × . The spatial resolution was 1 × 1 km^2^.

The data acquisition was performed with the Selenium package. The processing was based on the geopandas package^26^ using a geospatial vector layer for the district boundaries of Germany^32^. Each grid was processed to obtain the average value of the parameter over each district. We first used the function *within* to define a mask based on the grid centers contained in the district; we then applied this mask to the grid. In this method, called “central point rasterizing”^33^, each rectangle of the grid was assigned to a single district, the one that contained its center. The typical processing error was estimated to be about 1%, which agrees with the rasterizing error reported by Bregt et al.^33^; we consider that most likely this error is significantly less than the uncertainties of the grids themselves, caused by calculation, interpolation, and erroneous or missing observations.

#### Data structure

Our analysis was performed at the district level based on the annual infections, acquired by aggregating the weekly cases. From each monthly weather parameter, we created 24 records, for all months of the two previous years. Each observation in our dataset characterized one district in one year. Its target was acquired by transforming the annual incidence, as described in the following section. Each observation comprised all 168 available predictors from the weather parameters (7 parameters × 24 months), thereafter called “variables”. The notation for the naming of the variables follows the format Vx_<parameter>_<month>, where “Vx” can be V1 or V2 that corresponds to one or two years before, respectively; <parameter> is the abbreviation of the weather parameter (see previous subsection: “Weather data”); and <month> is the numerical value of the month, i.e., from 1 to 12.

The observations for combined districts retained the label of the rural district. For their infections and populations, we aggregated the individual values, and recalculated the incidence. For their weather variables, we assigned the mean values weighted by the area of each district.

### Target transformation

**Figure 5:**
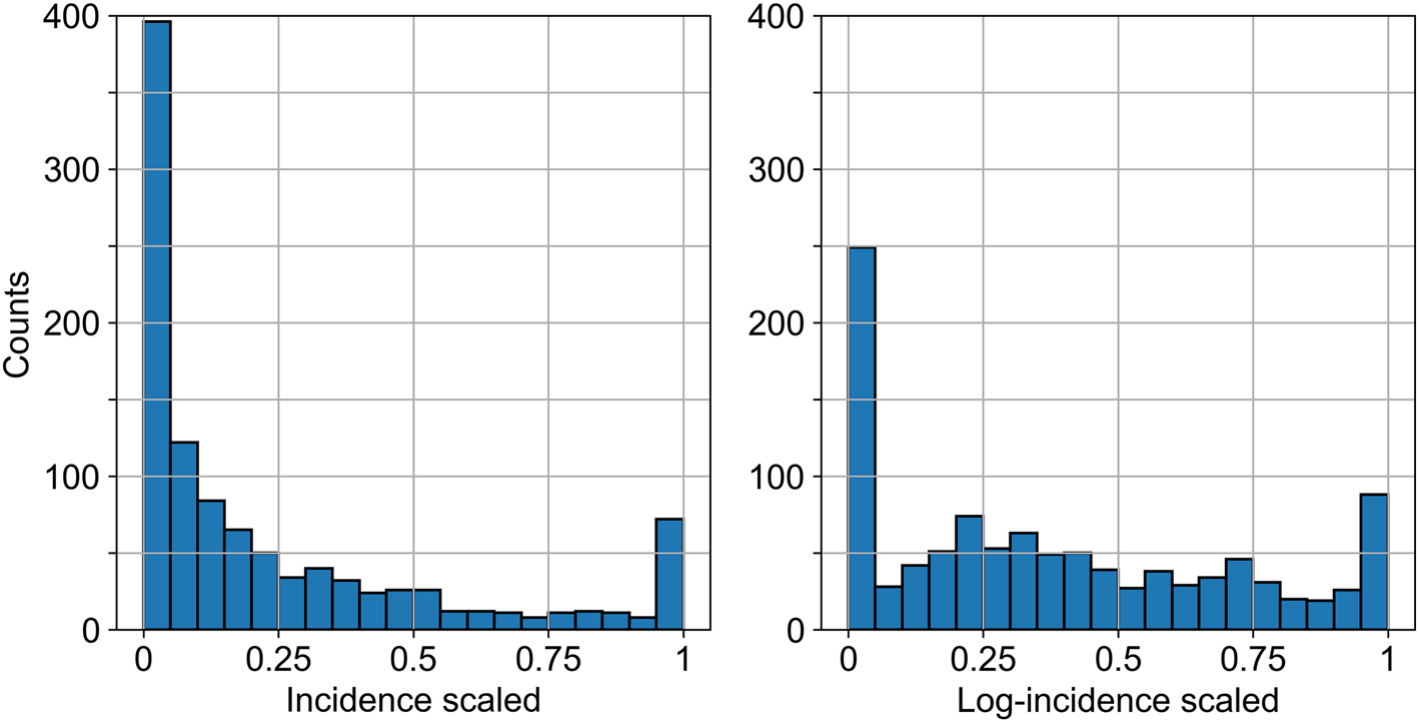
Histograms of the annual PUUV incidence from 2006 to 2021, scaled to its maximum value for each of the selected districts. Left: Raw incidence. Right: Log-transformed incidence, according to Eq. (6).

To consider the effects that drive the occurrence of high district-relative incidence, we discretized the incidence at the district level. The incidence scaled at its maximum value for each district showed extreme values for minima and maxima. About 49% of all observations were in the range [0, 0.1) and 8% in the range [0.9, 1] (Fig. 5). Therefore, we specifically selected to discretize the scaled incidence with two bins, i.e., to binarize it.

We first applied a log-transformation to the incidence values^34^, described in Eq. (6).

6 $${\text{Log - incidence}} = \log_{10} \left( {{\text{incidence}} + 1} \right)$$

The addition of a positive constant ensured a noninfinite value for zero incidence, with 1 selected so that the log-incidence is nonnegative, and a zero incidence was transformed into a zero log-incidence. This transformation aimed to increase the influence of nonzero incidence values; values that are not pronounced, but still hint at a nonzero infection risk. Its effect is demonstrated in the right plot of Fig. 5, where the positive skewness of the original data is reduced, i.e., low incidence values are spread to higher values, resulting to more uniform bin heights in the range [0.05, 0.95] after the transformation. Formally, in this case the log-transformation achieves a more uniform distribution for the non-extreme incidence values.

For the binarization, we performed unsupervised clustering of the log-transformed incidence, separately for each district, applying the function *KBinsDiscretizer* of the scikit-learn package^29^. Our selected strategy was the k-means clustering with two bins, because it does not require a pre-defined threshold, and it can operate with the same fixed number of bins for every district, by automatically adjusting the cluster centroids accordingly.

### Classification method

We concentrated only on those variable combinations that led to a linear decision boundary for the classification of our selected target. We selected support vector machines (SVM)^35^ with a linear kernel, because they combine high performance with low model complexity, in that they return the decision boundary as a linear equation of the variables. In addition, SVM is geometrically motivated^36^ and expected to be less prone to outliers and overfitting than other machine-learning classification algorithms, such as the logistic regression. For the complete modelling process, the regularization parameter C was set to 1, that is the default value in the applied *SVC* method of the scikit-learn package^29^, and the weights for both risk classes were also set to 1.

### Feature selection

Our aim was to use the smallest possible number of weather parameters as variables for a classification model with sufficient performance. To identify the optimal variable combination, we first applied an SVM with a linear kernel for all 2-variable combinations of the monthly weather variables from V2 and V1, i.e., 168 variables (7 weather parameters × 2 years × 12 months). Only for this step, the variables were scaled to their minimum and maximum values, which significantly reduced the processing time. For all the following steps, the scaler was omitted, because the unscaled support vectors were required for the final model. From the total 14,028 models for each unique pair ($\frac{168!}{2!\cdot \left(168-2\right)!}$), we kept the 100 models with the best F_1_-score, i.e., of the harmonic mean of sensitivity and precision, and counted the occurrences of each year-month combination in the variables. The best F_1_-score was 0.752 for the pair (V1_Tmean_9 and V2_Tmax_4); and the best sensitivity was 83% for the pair (V2_Tmax_9 and V1_ST_9).

The year-month combinations with more than 10% occurrences were: V1_9 (September of the previous year, with 49% occurrences), V2_9 (September of two years before, with 12%) and V2_4 (April of two years before, with 10%). To avoid sets with highly correlated variables, we formed 3-variable combinations, with exactly one variable from each year-month combination (threefold Cartesian product). From the total 343 models (7^3^ combinations, i.e., 7 weather parameters for 3 year-month combinations), we selected the model with the best sensitivity and at least 70% precision, i.e., the variable set (V2_ST_4, V2_SD_9, and V1_ST_9). We consider that the criteria for this selection are not particularly crucial; and we expect comparable performance for most variable sets with a high F_1_-score, because the variables for each dimension of the Cartesian product were highly correlated. The eight variable sets with at least 70% precision and at least 80% sensitivity are shown in Supplementary Table 2.

The SVM classifier has two hyperparameters: the regularization parameter C and the class weights. By decreasing C, the decision boundary becomes softer and more misclassifications are allowed. On the other hand, increasing the high-risk class weight, the misclassifications of high-risk observations are penalized higher, which is expected to increase the sensitivity and decrease the precision. The simultaneous adjustment of both hyperparameters ensures that the resulting model has the optimal performance with respect to the preferred metric. However, in order to avoid overfitting, we considered redundant a further model optimization with these two hyperparameters. For completeness, we examined SVM models for different values of the hyperparameters and found that the global maximum for the F1-score is in the region of 0.001 for C and 1.5 for the high-risk class weight. Our selected values C = 1 and high-risk class weight equal to 1 give the second best F1-score, which is a local maximum with comparable performance, mostly insensitive to the selection of C from the range [0.2, 5.5].

The addition of a fourth variable from V1_6 (June of the previous year) resulted in a model with higher sensitivity but lower precision and specificity (for V1_Pr_6). The highest F1-score was achieved for the quadruple (V2_ST_4, V2_SD_9, V1_ST_9, V1_Pr_6). Because of the increased complexity without significant improvement in the performance, we considered unnecessary a further expansion of our variable triplet.

## Supplementary Information

Supplementary Information.

## Data Availability

The data that support the findings of this study are available from the corresponding author upon reasonable request.

## Code availability

Information about the used software and packages is provided in the main manuscript. The code that supports the findings of this study is available from the corresponding author upon reasonable request. Supplementary Information is available for this paper.
