# Supplementary material for: Machine learning identifies straightforward early warning rules for human Puumala hantavirus outbreaks
Source: Sci Rep. 2023 Mar 3;13:3585. doi: 10.1038/s41598-023-30596-x (PMC9984366; doi:10.1038/s41598-023-30596-x)
Supplement: Supplementary file 1 — Supplementary Information. [file 41598_2023_30596_MOESM1_ESM.pdf]

Machine learning identifies straightforward early warning rules for human Puumala hantavirus outbreaks

Supplementary information

Orestis Kazasidis*, Jens Jacob
Julius Kühn Institute (JKI) – Federal Research Centre for Cultivated Plants, Institute for Plant Protection in Horticulture and Forests / Institute for Epidemiology and Pathogen Diagnostics, Rodent Research, Toppheideweg 88, 48161 Münster, Germany
Corresponding author: orestis.kaza@gmail.com

Supplementary Table 1: Combined urban and rural districts

Supplementary Table 1: Combinations of urban and neighboring rural districts to be included in the model. The resulting pooled data retained the label of the rural district.

Federal state	Urban district	Rural district
Baden-Württemberg	Heidelberg	Rhein-Neckar-Kreis
	Heilbronn	Heilbronn
	Karlsruhe	Karlsruhe
	Pforzheim	Enzkreis
	Ulm	Alb-Donau-Kreis
Bavaria	Aschaffenburg	Aschaffenburg
	Passau	Passau
	Schweinfurt	Schweinfurt
	Würzburg	Würzburg
Hesse	Kassel	Kassel
Lower Saxony	Osnabrück	Osnabrück
Thuringia	Eisenach	Wartburgkreis

Supplementary Table 2: Support Vector Classifier with three-variable combinations

We formed 343 models, based on three year-month combinations: April and September of two years before (V2_9 and V2_4), and September of the previous year (V1_9). Supplementary Table 2 shows the variable sets with at least 80% sensitivity and at least 70% precision. We selected the variable set (V2_ST_4, V2_SD_9, and V1_ST_9) that had the best sensitivity. The variable V2_SD_9 (sunshine duration in September of two years before) was contained in most sets, most commonly in combination with the variable V1_ST_9 (soil temperature in September of the previous year).

Supplementary Table 2: The variable-triplets that led to a classification model with at least 80% sensitivity and at least 70% precision, in order of decreasing sensitivity. The performance metrics correspond to an SVC with a linear kernel, with C=1 and equal class weights.

Three-variable set	Sensitivity	Precision	F_1-score
(V2_ST_4, V2_SD_9, and V1_ST_9)	0.8476	0.7140	0.7751
(V2_Tmean_4, V2_SD_9, and V1_ST_9)	0.8369	0.7313	0.7805
(V2_Tmax_4, V2_SD_9, and V1_ST_9)	0.8316	0.7352	0.7804
(V2_Tmean_4, V2_SD_9, and V1_Tmax_9)	0.8316	0.7100	0.7660
(V2_Tmin_4, V2_SD_9, and V1_ST_9)	0.8262	0.7023	0.7592
(V2_Tmax_4, V2_SD_9, and V1_Tmax_9)	0.8235	0.7113	0.7633
(V2_Tmax_4, V2_Tmean_9, and V1_ST_9)	0.8182	0.7338	0.7737
(V2_Tmean_4, V2_SD_9, and V1_Tmean_9)	0.8128	0.7308	0.7696

Supplementary Note 1: A close inspection of underestimations regarding recent classifier values in Northern Germany

From the total 57 false negatives, 35 originated from the federal states of Lower Saxony and North Rhine-Westphalia. Almost half of these observations (15) were from recent non-outbreak years, i.e., from 2016, 2018, and 2020. Since no false negatives originated from the district of Osnabrück that had the most total infections in these two federal states, we hypothesize that the false negatives of our classifier were not due to a local outbreak, but rather were caused by an increase in the PUUV-baseline in specific districts, i.e., by increased monitoring and reporting during a low-risk year. This hypothesis is supported by the trend shown in Supplementary Note 1 – Figure 1 for the incidence time series in the districts of Borken, Emsland and Steinfurt, especially for their values in 2016 and 2020, when the presumed baseline increase occurred simultaneously in all three districts.

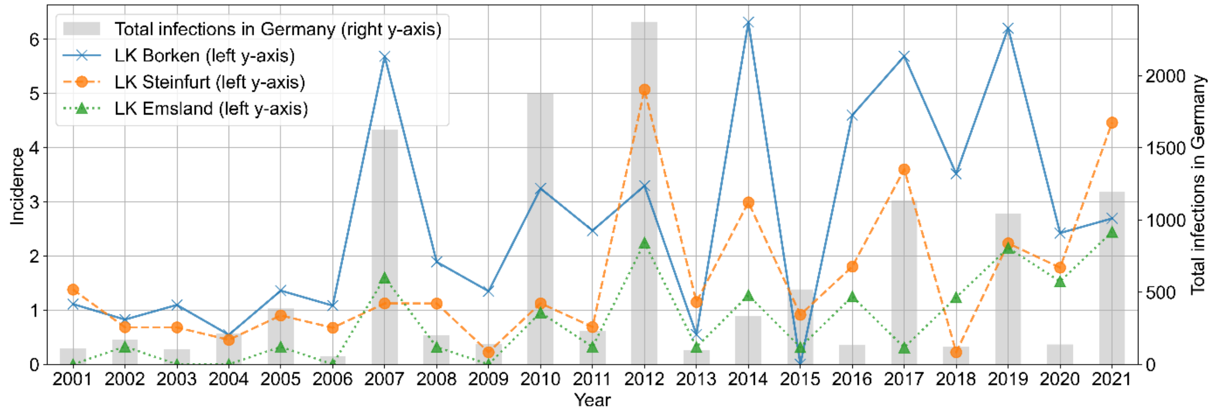

Supplementary Note 1 – Figure 1: **PUUV-baseline increase in North-Western Germany.** The time series of the incidence in the districts of Borken (left y-axis, x-markers, solid blue line) and Steinfurt (left y-axis, o-markers, orange dashed line) from North Rhine-Westphalia, and Emsland (left y-axis, triangle-markers, dotted green line) from Lower Saxony; from 2001, when hantavirus disease became notifiable in Germany, until 2021. In all three districts, an unusually high incidence was reported in 2016 and 2020. The grey bars (right y-axis) indicate the total PUUV-infections in Germany.

Another possible explanation is that the PUUV-season may start earlier in Northern Germany in comparison to the other PUUV-clusters, i.e., already from early autumn of the year preceding an outbreak. This seems plausible particularly in the district of Bentheim, where the annual incidence in 2016 and 2018 was the maximum ever recorded (7.4 with 10 infections, in both years). The majority of these infections was reported after the 36th week of each year, i.e., after mid-September. Specifically, as shown in Supplementary Note 1 – Figure 2, four infections were reported before the 36th week, and six infections afterwards. Both 2016 and 2018 were followed by years that the model classified as outbreaks for the majority of the districts, including the district of Bentheim.

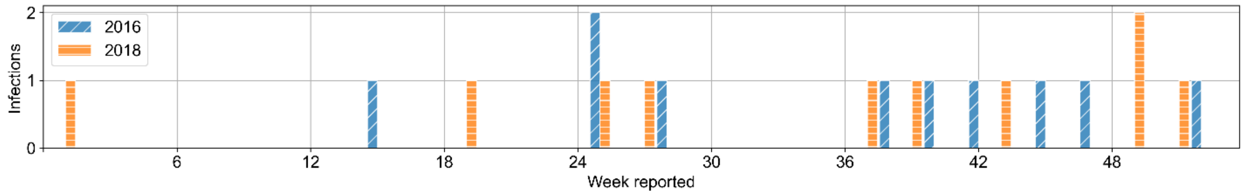

Supplementary Note 1 – Figure 2: **Weekly infections in the district of Bentheim for 2016 and 2018.** The weeks for all reported PUUV-infections in 2016 (blue bars with diagonal hatch) and 2018 (orange bars with horizontal hatch).
